# Proposal for a Monitoring Concept for Veterinary Medicinal Products with PBT Properties, Using Parasiticides as a Case Study

**DOI:** 10.3390/toxics6010014

**Published:** 2018-02-09

**Authors:** Jörg Römbke, Karen Duis

**Affiliations:** ECT Oekotoxikologie GmbH, 65439 Flörsheim am Main, Germany; k.duis@ect.de

**Keywords:** VMP, persistence, bioaccumulation, toxicity, dung organisms, post-authorization monitoring, field study

## Abstract

The aim of this work is to prepare a proposal for the post-authorization monitoring (PAM) of veterinary medicinal products (VMP), in particular parasiticides. Such a monitoring might especially be useful for parasiticides identified as Persistence Bioaccumulation Toxicity (PBT) substances, i.e., chemicals that are toxic (T), persist in the environment (P) and bioaccumulate (B) in food chains and, thus, pose a hazard to ecosystems. Based on a literature search, issues to be considered when performing such a PAM are discussed (e.g., residue analysis, compartments to be included, selection of organisms and the duration of monitoring studies). The outcome of this discussion is that—and despite that there are huge challenges in detail (e.g., in terms of analytical chemistry or taxonomy)—the technical performance of such a PAM is not the main problem, since most of the chemical and biological methods to be used are well-known (partly even standardized) or could be adapted. However, it is very difficult to define in detail where and when a monitoring should be performed. The main problem is to link exposure to effects of a certain parasiticide in a way that any impact can directly be related to the use of this parasiticide. Therefore, a “Targeted Environmental Monitoring” (TEM) is proposed, which is essentially a combination between a field study and a PAM.

## 1. Introduction

In order to assess the potential risk of veterinary medicinal products (VMP), and in particular, parasiticides, to the environment within authorization procedures guidelines have been published by the International Cooperation on Harmonization of Technical Requirements for Registration of Veterinary Medicinal Products (VICH), a trilateral program to harmonize technical requirements for VMPs in Europe, Japan, and the United States [1] and the Committee for Medicinal Products for Veterinary Use (CVMP) of the European Medicines Agency. VMPs, and in particular, parasiticides (mainly belonging to the avermectins and, to a lesser extent, to the milbemycins and spinosyns), (all together known as macrocyclic lactones (MLs)) are regularly used world-wide. They are effective against a wide range of parasites, especially those affecting farm animals (e.g., cattle, sheep, horses etc.) and have been found in different environmental compartments (water, sediments, soils) [2]. In particular the ivermectin is well known to affect also non-target organisms [3], which often belong to the same taxonomic group as the parasites [4]. Thus, there is robust evidence that these parasiticides cause environmental risks [5].

Environmental risk assessments (ERAs) are performed in a tiered approach: in Phase I [6] general aspects regarding use and exposure are evaluated, and in Phase II tests are required [7]. A Phase II ERA including tests on dung fauna is always required for parasiticides used for the treatment of pasture animals.

The risk assessment procedure described in the respective guidelines focuses on the quantitative determination of the environmental risk of a VMP—i.e., the comparison of predicted exposure and effect concentrations. However, it is also important to identify those VMPs, which—because of their inherent properties—may pose a hazard to the environment [8]. In [9] it is laid down how a PBT assessment has to be performed.

PBT substances are toxic (T), persist in the environment (P) and bioaccumulate (B) in organisms and, thus, can pose a hazard to human health and ecosystems [10]. One suitable approach to this problem is to address the PBT properties in a benefit-risk assessment in accordance with Commission Directive 2009/9/EC [11] amending Directive 2001/82/EC [12]. Potential outcomes of such a benefit-risk assessment would be no authorization, or a conditional marketing authorization for the specific VMP. In the latter case, a possible condition could be to impose a post-authorization monitoring (PAM) as mentioned in [9]: “Targeted sampling/post-marketing monitoring in the environment following treatment [...] could be envisaged to allow measurement of effectiveness of risk mitigation measures (RMM). Although experience with this is limited at present, availability of monitoring data would contribute to a better understanding of the risks”. Monitoring activities (e.g., determination of the actual environmental exposure to the respective VMP) could be required on a case-by-case basis as part of a risk management plan (RMP). Such monitoring would be in line with the post-marketing surveillance (PMS), which is an important tool of pharmacovigilance. However, details of such a PAM for VMPs have not yet been established.

Before starting a PAM for VMPs, the aim of such a monitoring has to be clarified, i.e., the respective protection goals need to be established. According to [7], the aim of the ERA for VMPs is to protect ecosystems and, in this context, to evaluate the potential to affect non-target species in the environment, including both aquatic and terrestrial species. Similar objectives are defined in other national legislations, e.g., the German Federal Soil Protection Act [13], or European documents on chemical risk assessments [14,15]. For example, Regulation (EC) No. 1107/2009 addressing plant protection products (PPP) defines general protection goals that aim at protecting biodiversity and ecosystems [16]. In line with the ecosystem service approach [17], both the structural diversity (i.e., the species composition) as well as the functional diversity (i.e., the benefits) of organism communities have to be protected. However, the protection goals for VMPs have not been specified accordingly. The discussion in this article, will focus on the protection of the diversity of dung insect communities, in particular, their functions (most prominently, dung degradation), since it is well known that these organisms are strongly affected by VMPs [2,18]. T However, these protection goals have not been laid down in the European VMP legislation so far.

The aim of the work described here is to prepare a proposal for the post-authorization monitoring (PAM) of VMPs, particularly parasiticides, either having been identified as causing high environmental risks (i.e., those with a risk quotient >> 1) or are classified as PBT substances. Monitoring activities should focus on:

- The detection of these parasiticides in the selected environmental compartments, i.e., in soils, surface waters including suspended particles and sediments;
- The accumulation of the parasiticides in organisms living in these compartments;
- The effects of the parasiticides on exposed organisms, mainly arthropods, which are most likely to be affected by parasiticides, in particular dung flies and beetles, but also soil (e.g., Collembola) or sediment (e.g., insect larvae) insects.

Finally, it should be noted that examples are mainly taken from the terrestrial compartment (dung and soil), because exposure is most likely here and the amount of available data is highest.

## 2. Methodological Approach

A literature search was performed using the following search terms in various combinations: parasiticide, monitoring, bioaccumulation, effects, exposure, fate, dung, soil, sediment, surface water. Basic compilations on the usage of monitoring methods (including theoretical considerations), for both exposure and effect endpoints, were also included in this search. In this context, several guidance documents describing field methodologies were checked for useful information, e.g., regarding the sampling of soil organisms or the measurement of organic matter degradation [19,20] . Relevant aspects related to the post-authorization of parasiticides were compiled based on personal experience, e.g., from field studies performed with ivermectin [21,22], or information from available literature [23]. In addition, experiences from other legal frameworks such as the registration of pesticides [16,24], or the environmental risk assessment of genetically modified organisms (GMO) [25] were considered.

For each parasiticide to be studied in a PAM, the following information has to be considered when planning the practical field work:

- its physico-chemical properties;
- its application rate and frequency for the treatment of farm animals;
- its concentrations and fate in different environmental compartments, including predicted environmental concentrations (PECs);
- available analytical and extraction methods (including, e.g., detection limits);
- its excretion pathway, excreted amounts and major metabolites;
- results of bioaccumulation and ecotoxicity tests with the parasiticide.

## 3. Results

### 3.1. Short Overview on Existing Monitoring Concepts

The term “monitoring” is used in different ways. Often, it is defined as “intermittent (regular or irregular) surveillance carried out in order to ascertain the extent of compliance with a predetermined standard or the degree of deviation from an expected norm” [26].

Generally, monitoring can be described as a systematic measurement of variables and processes in a standardized manner at intervals over time related to a specific reason. There are many different types of monitoring, e.g.,:

- Background monitoring (i.e., determination of environmental reference conditions);
- Impact monitoring (i.e., measurement of the effects of anthropogenic activities);
- Trend monitoring (i.e., identification of long-term and/or large-scale changes).

In addition, Hommen et al. [24] distinguished three different monitoring approaches:

- Ecotoxicological monitoring: determination of the toxicity of chemicals in the respective environmental compartment, either by exposing organisms directly in the field (e.g., in cages) or by evaluating water, sediment or soil samples brought from the field to the laboratory;
- Chemical monitoring: measurement of the concentrations of specific chemicals in different environmental compartments (so far, most often in surface waters);
- Ecological monitoring: detection of effects on biological entities (e.g., populations, species or communities) in the field and their assessment in relation to site-specific environmental parameters, including concentrations of chemicals.

In general, these three approaches could (and should) be combined. However, in case of the PAM for parasiticides, focus will be placed on the latter two approaches. Please note that monitoring of the bioaccumulation of parasiticides could belong to all three approaches.

For about 100 years, the diversity and abundance (partly also the biomass) of organisms living in surface waters and sediments have been used to classify the biological quality of the respective environmental compartments. One well-known example is the Saprobian system, which indicates the water quality based on the presence or absence of certain species with known preferences [27]. National (partly regional) keys are available for the most important freshwater organisms as well as basic ecological information regarding the factors governing their distribution [28,29,30]. Since more than 90 years, it is known that the composition of plant communities is a very good endpoint to describe and assess the biological quality of terrestrial sites [31]. However, data on the ecology and taxonomy of dung and soil organisms are often lacking, even in “well-studied” regions such as Central Europe [21,32]. Exceptions are the rather few ecotoxicological studies with dung organisms performed within the context of parasiticide authorization [33]. Such studies are often performed in a comparable way as regulatory field studies focusing on the effects of pesticides on soil organisms, especially earthworms. For about 20 years, the latter have to be performed according to international guidelines [34]. Available biogeographical or ecological studies usually only provide the name of the species as well as the name of the sampling site, i.e., data on the characterization of the sampling site, the specific exposure conditions or details of the sampling methodology are often not provided. Therefore, it is of urgent importance to get an overview of the “normal” (or reference) range of the species distribution and the functional roles of dung and soil organisms. Otherwise, any monitoring approach will fail short, since it will not be possible to decide whether data from the evaluated site indicate a good or bad biological quality. In order to use such an approach in a practical way, it is necessary to collect the ecological and biogeographical data on the respective organism groups in an accessible database. This has been done for soil organisms in the German Edaphobase [35]; similar activities are known from France [36], The Netherlands [37], and Great Britain [38]. No such activities are known for dung organisms, but the need for such work is acknowledged in Switzerland [39].

### 3.2. Presentation and Evaluation of Monitoring Data

**Figure 1:**
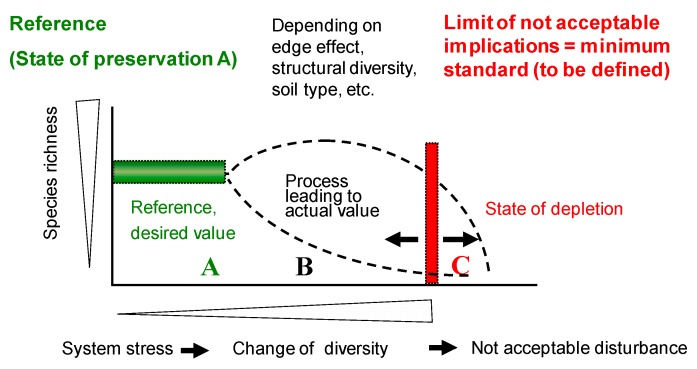
Derivation of references values in relation to a desired range (e.g., in terms of species number, community composition etc.): A, B and C represent different preservation states depending on the level of stress (e.g., soil contamination), reproduced with permission from [32]. Copyright Soil Organism, 2013.

Hommen et al. (2004) [24] discussed general pros and cons of the use of monitoring approaches as part of ERAs of pesticides, especially in view of the high variability of all ecological endpoints measured in the field. In order to overcome this problem different endpoints should be evaluated applying a weight-of-evidence (WoE) approach. The determination of both chemical concentrations and biological effects at the same site and point in time will help to identify the cause of observed effects. In contrast to planned field tests, monitoring usually does not contain independent control sites, i.e., sites with the same properties as the sites under assessment, but without contamination. There are some possibilities to overcome this problem:

- Identification of an uncontaminated site close to the monitoring sites, with both sites being as similar as possible in their properties. This is possible but very difficult, especially because it is often unknown, which factors influence the occurrence, diversity or abundance of certain organisms at a given site.
- A more robust approach is the identification of a number of uncontaminated sites with the same land-use (for parasiticides mainly grasslands) in the same biogeographic region, e.g., as defined by [40]. By statistical evaluation of such data (and with a suitable number of sites) reference ranges for different organism groups and endpoints could be defined (see Figure 1).

The basic assumption is that in an uncontaminated site, a certain diversity of an organism community (measured as species richness) is found (state A). Stress, e.g., caused by a parasiticide is increasing the “normal” changes in diversity (state B): Depending on various site-specific factors, the diversity may decrease (either immediately or after some time). From a regulatory point of view, the main problem is to identify the point when such a change is considered not acceptable, i.e., when the state of depletion starts (state C).

**Figure 2:**
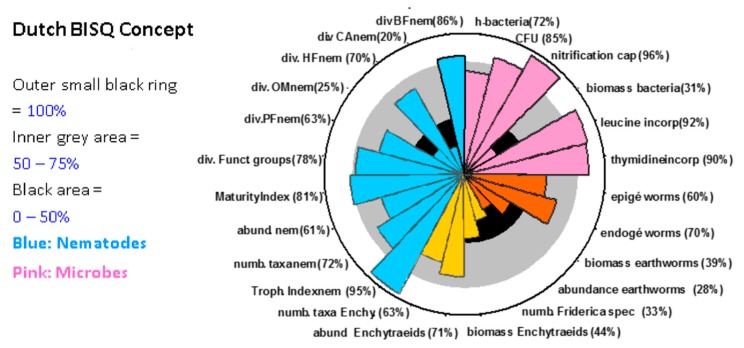
Results of a soil biological monitoring at a Dutch conventional farm in relation to reference data gained at an ecologically managed farm ([44], modified). Results for each organism group and endpoint (e.g., abundance, or biomass) are given in percent of the reference value for the same site type (100%, shown as outer black line). A difference of 25–50% (grey area) is classified as being of low and a difference of >50% as being of large concern. Abbreviations: abund = Abundance; BF = Bacterial-feeding nematodes; CAnem = Carnivorous nematodes; CFU = colony-forming units; div = diverse; Enchy = Enchytraeids; Funct = functional group; h-bacteria = Heterotrophic bacteria; HFnem = Hyphal-feeding nematodes; incorp = Incorporation; nem = Nematoda; numb = Number of species; OMnem = Omnivorous nematodes; PFnem = Plant-feeding nematodes; Spec = species; troph = trophic.

There are different possibilities to compile and present monitoring results. The most basic ones are tables including e.g., names of species and their abundance, which form the basis for indices or indicator values summarizing the characteristics of the whole organism community. The Ellenberg’s values are the best-known example, showing the dependency of plant species (or the whole plant community) of factors such as temperature, soil pH, or soil moisture [41]. In the context of the implementation of the Water Framework Directive (WFD) [42] the usage and importance of such indices in various European countries increased [43]. These approaches have often been used in environmental monitoring programs due to their easy interpretation (especially when grouping them in a limited number of classes ranging from “very good” to “very bad”). However, in relation to the effort of getting these indices or indicator values (i.e., in terms of taxonomic determination of individual species) a lot of information is lost when only a single number is reported. A graphical way of presenting monitoring data from a site is the Amoebae approach (Figure 2; [44]). This approach has been used in a Dutch monitoring program assessing the biological quality of soils. Results of a PAM for parasiticides could be evaluated using such approaches.

### 3.3. Veterinary Medical Products (VMPs), Especially Parasiticides

For more than 40 years, it is known that VMPs, in particular parasiticides, can pose a risk to the environment [2,45,46]. Especially their high toxicity to terrestrial invertebrates (mainly arthropods living in or on dung of farm animals) and aquatic invertebrates (mainly Daphnia) is well documented [2,3,47]. Other invertebrates are usually less affected—a fact that can partly be explained phylogenetically [4], partly by the lack of data for many of these groups. Since both target and non-target organisms often belong to the same taxonomic group (e.g., insects such as flies) parasiticides potentially affect non-target organisms, which are responsible for important ecosystem functions (and thus ecosystem services) such as the degradation of dung ([48,49,50]. In addition, any direct effect on dung beetles may also have an impact on their predators such as owls [51].

Parasiticides cannot only be toxic and bioaccumulative but also persistent in the environment. Transformation of the well-known parasiticide ivermectin in soil, for instance, was investigated according to [52]. The results indicate that dissipation half-lives (DT50) in soil ranged from 16 to 1520 days, depending on soil type, sorption capacity, temperature, and oxygen availability [3,53]. Accordingly, a range of DT50 values (of <0.25 to 127 days) were determined in water-sediment systems, depending also on sediment properties [3,54].

Data on bioaccumulation of parasiticides are scarce, even for the well-studied substance ivermectin. This is related to its log Kow of 3.2, which is lower than the value of ≥4 triggering a bioconcentration study for a parasiticide [7]. However, bioaccumulation of ivermectin was studied in the oligochaete worm *Lumbriculus variegatus* in artificial and natural sediments [55]. Depending on the sediment characteristics (mainly the content of organic matter), the biota/sediment accumulation factor (BSAF28 d) varied between 1.9 and 5.5, indicating a potential for bioaccumulation.

### 3.4. Issues to Be Considered for a PAM of Parasiticides

In the following, issues relevant for a PAM of parasiticides are presented focusing on the scientific suitability and technical practicability of a PAM.

#### 3.4.1. Study Design

Depending on the objectives and endpoints of a PAM the sampling design will differ. Since the statistical power depends on the way the sampling is performed (e.g., how many replicates and time points are used) a PAM has to be planned very carefully (see also [20,56]).

#### 3.4.2. Environmental Compartments and Residue Analysis

Robust and reliable data on environmental concentrations of the active substance of the parasiticide in surface waters including suspended particles, sediments, soils and dung should be determined using validated methods. Based on the measured concentrations maximum values (peaks) and time-weighed-averages should be derived. In order to include the aspect of bioavailability the active substance could be measured in both the respective bulk (dung, soil or sediment) phase and the pore water. This approach is well-known for soils and sediments, but has not been used in dung so far [57]. Dung is usually degraded within a few weeks to months at the latest under central European conditions. Therefore, its handling (including data interpretation) is more difficult in comparison to the other three compartments. However, since the degradation of dung is probably the most important ecosystem service provided by the dung organism community it should be monitored [18].

#### 3.4.3. Bioaccumulation

Assuming that a parasiticide with PBT properties has been detected in the environment, it has also to be checked whether this substance accumulates in biota. Large invertebrates feeding on dung and/or living in soil (e.g., beetle larvae, earthworms) or sediments (e.g., lumbriculid worms) might be suitable for this purpose. In addition, predatory organisms such as owls could be included [51], but it is not well known which organisms feed to what extent on dung organisms. However, the use of vertebrates in monitoring studies should be restricted as much as possible due to animal protection reasons. So far, there is only one guideline available addressing bioaccumulation in the field [58]. It covers the accumulation of chemicals in deep-burrowing (=anecic) lumbricid earthworms. This guideline is used mainly for a long-term environmental monitoring and assessment program, the German Environmental Specimen Bank [59]. In this program, the accumulation of 28 pharmaceuticals in fish has been monitored, however, parasiticides are not amongst them.

#### 3.4.4. Organisms to Be Sampled

The selection of organism groups to be monitored is relatively easy: the main criteria are sensitivity towards the specific parasiticide (based on information from ecotoxicological tests), occurrence (based on biogeographical and ecological information from previous monitoring exercises or studies) and functional importance. Species on Red Lists have to be excluded due to their high protection status. For sediments, arthropod species such as dipteran larvae (e.g., Chironomidae) should be selected due to their sensitivity [30,60]. In dung, flies (e.g., Sepsidae) and beetles (i.e., Aphodiidae, Scarabaeidae) have to be considered. For example, Jochmann et al. [61] published a list of organism groups (mainly families), which can be considered as typical for dung organism communities in temperate grasslands. The selected groups will differ depending on the ecotoxicological properties and usage patterns of the parasiticide to be monitored. However, there are still deficiencies in our knowledge regarding the regional distribution as well as the classification of this information with regard to “ecological” or “trait groups”. Reference values (or ranges) for individual species or communities are only available for the aquatic environment [42]; for soil they are in development. Methods already successfully used in research projects should be standardized internationally (e.g., genetic methods such as barcoding for species identification; [39,62]. Such genetic methods rely on databases containing both sequence data and robust morphological information defining individual species. Only by bringing both sets of information together, the biological and ecological knowledge of the last 50 years can be used.

#### 3.4.5. Endpoints to Be Measured

Effects on biodiversity are assessed by determining species number, species composition and abundance of organisms in all compartments where the parasiticide does/could occur. In order to get a better overview on the functional consequences of changes of biodiversity (e.g., the loss of certain species), it might be useful to evaluate not only the whole community but also specific indicator species, i.e., those which are functionally highly important. For example, in soil this could be the epigeic earthworm *Lumbricus castaneus*, which is known to feed regularly on dung (Adam Scheffczyk, personal observation) and thus could be exposed to parasiticides. Some of the large deep-burrowing scarabaeid beetles have a similar important role [63].

In the last decade, species determination as well as the definition of functional or ecological groups (best known: epigeic, endogeic, and anecic earthworms [64] in aquatic and soil compartments has been improved considerably. However, there is still a gap in ecological or biogeographical knowledge for many groups of dung organisms. As mentioned above, many biogeographic and ecological data are given without supporting information on the site properties [65,66,67,68]. Standard methods and standard operation procedures should be used when performing monitoring activities in the field [19,69].

In summary, there is a huge amount of experience, which can be used to identify endpoints for the effect monitoring of organisms. Abundance and structural diversity are the most obvious choices. The problem of the effect monitoring is the potentially high effort needed.

#### 3.4.6. Duration of the Monitoring

The duration of a monitoring study depends very much on the usage of the respective parasiticide in different regions and farm animals as well as on the distribution of potentially affected non-target communities in time and space.

In addition, the persistence and thus the potential duration of effects of the parasiticide has to be taken into account. A study duration of several years (e.g., 3 years) with at least two measurements per year is recommended. Experiences made in other areas of post-registration monitoring (e.g., the German Environmental Specimen Bank [70]) have to be evaluated before providing more detailed recommendations. The respective sampling scheme will probably differ in the individual compartments.

#### 3.4.7. Target Farm Animals

Obviously, monitoring of a parasiticide in the environment does only make sense, if there is a probability of contamination in a certain region and at a certain point-in-time, which leads to a concern. Depending on the regional and seasonal conditions (e.g., the occurrence of parasites) and the farm animals to be treated, the recommended route of administration, dose and frequency of administration will vary. In Europe, cattle, horses and sheep are most relevant farm animals, since they are the most common and probably most frequently treated farm animals. Other species (e.g., goats) may be relevant in some cases. Finally, the farm animals used in a PAM should not only be selected on a worst-case scenario based on the highest PEC_Dung_, since dung organisms exposed to a lower concentration of the parasiticide in another type of dung might be more sensitive. Since dung of the different farm animals is—at least partly—inhabited by different dung organism communities, it should be carefully checked whether those found in the dung of other farm animals are protected too, when only dung of the most commonly treated animal is monitored.

#### 3.4.8. Target and Control Sites

In contrast to planned field studies, where a control is always defined beforehand, usually no control site with comparable properties (e.g., in terms of climatic conditions, soil properties or non-target organism communities) is available in close distance to the target site. In addition, the occurrence of other (mainly anthropogenic) stressors has to be taken into account (e.g., pesticides) Target sites are mainly pastures used regularly for keeping farm animals (mostly cattle), which were treated at least once (more often several times, according to the occurrence of parasites) with a parasiticide. Ideally, a control site would be a pasture with similar site and soil properties and ecological conditions (e.g., in terms of the composition of non-target organism communities), which has also been used for farm animal grazing but has no history of parasiticide applications. In any case, the treated and control sites should belong to the same biotope type [71]. Biotope types are defined in hierarchical way, starting on the highest level with large (and heterogeneous) land use types such as grasslands. On the second level, grasslands are divided according to their usage and soil properties. It is improbable that comparable pastures with and without parasiticide treated farmland animals are available in the same region at the same time. Theoretically, such a site could be found on an organic farm, however, even on organic farms parasiticide are used, mainly for reasons of animal welfare considerations taking priority over environmental concerns [72]. The same problem is also relevant for adjacent non-target areas, e.g., in surface waters (e.g., after treated cattle has used them for defecating or cooling).

#### 3.4.9. Conclusions Regarding the PAM for Parasiticides

- The performance of a PAM with parasiticides is technically not a huge problem. Most of the methods to be used are well-known (partly even standardized), or could be adapted.
- However, it is very difficult to define in detail where and when the monitoring should be performed. The main problem is to link parasiticide exposure with effects in a way that any impact can directly be traced back to a specific parasiticide.
- In order to overcome this problem, the PAM for parasiticides has to be modified as described in the next section.

## 4. Discussion: Approach for a Targeted Environmental Monitoring (TEM) of Parasiticides

One possibility to overcome the problems described in the previous section would be to perform a long-term field study under farm conditions (i.e., a targeted environmental monitoring, TEM).

### 4.1. Control and Reference Sites

A TEM should be performed at sites (=farms), where the parasiticide under consideration has been used to treat farm animals (i.e., the treatment regime and the number of treated farm animals per area are known). Such sites could be identified based on a survey made with farmers in an area, where the respective farm animals are commonly treated with the considered parasiticide. The treated sites should cover the various compartments, which are potentially at risk (e.g., a pond or small stream should be close). At present, it is not possible to determine how many sites would be needed to obtain results that are sufficiently representative for a specific treatment regime and region. The need for an untreated control could be handled in different ways:

- If the TEM is performed at sites without strong parasite pressure, one group of farm animals could be treated with the parasiticide, while another group could remain untreated. Both groups would be kept at the same site. This approach is similar to higher-tier field studies [21].
- Ideally, sites could be selected that are farmed conventionally (test) or organically (control), preferably as pairs in the same region.
- A detailed history of the individual sites including information on the treatments should be available. Theoretically, control sites would be close to the test sites but without treatment with the parasiticide under evaluation (or with similar mode-of-action), for at least e.g., five (?) years (as it is done when testing plant protection products). However, as mentioned above it is improbable to find such control sites.

In case control sites are not available, the “reference approach” should be used. It is defined as follows: pasture sites are selected, which have not been used for the treatment of farm animals with parasiticides but belong to the same biotope type as the farm sites where treatments have been performed. Environmental conditions (i.e., climate, vegetation etc.) should be similar, assuming that in such a case also the organism communities are similar. These reference sites could be located close to the treated site (but this is not mandatory). It is assumed that at least the invertebrate communities in surface water, sediment and soil are similar when these sites belong to the same biotype [37]. For dung organism communities, the situation is probably similar, considering the long history of livestock farming in most parts of Europe. Average values for such reference sites (e.g., the abundance of specific species or the diversity of a specific community) are used as a reference normal operating range) in order to evaluate the monitoring results gained at treated sites. Regarding all other aspects of the practical performance, a TEM has to address the same issues as discussed in the previous chapter (e.g., validated and preferably standardized methods should be used).

### 4.2. Evaluation of Any Monitoring of Parasiticides

In the context of the post-registration monitoring of PPPs, [73] compiled a number of criteria that should be checked when evaluating its performance and results. The detailed documentation of the design and the performance of the study (including the identification of reference sites) is most important. In addition, a very close co-operation with the farm owners is crucial to get robust data regarding the actual administration of the test substance as well as historical data on the usage of parasiticides. Data from different scales of investigation (e.g., laboratory tests) or other study sites should be considered to allow for the interpretation of the ecological relevance of differences found between tests sites and control/reference sites.

When assessing the results of a PAM, the biggest problem is always the lack of a control. In contrast to a planned and controlled field study (e.g., an earthworm field study that is often required as part of the ERA of pesticides [34]) often no controls are available. The evaluation whether a measured effect is significant or not is depending on reference values (or ranges) which have to be established beforehand. For biological endpoints, comparable approaches are known for sediments with regionally different values [42]. Similar proposals for soils were developed in Germany and The Netherlands [32,37]. Within a recent research project, the database Edaphobase was established for the derivation of such biological soil values for selected organism groups such as Lumbricidae or Collembola (at least for Germany) [35].

## 5. Open Issues Needing Further Research

Generally, data on the use of parasiticides (e.g., prescribed amounts, frequency of usage) from well-described sites (i.e., geographical coordinates, dates, soil and site properties) are missing. A database for the EU or different countries would be helpful. Data on the accumulation of parasiticides in potentially exposed non-target organisms are lacking. In addition, there are few data on species, which might feed on dung organisms. In contrast to organism communities in sediments, surface waters and partly in soil, insufficient information is available on the structure and population dynamics of dung and soil organism communities. These data are required as basis for the reference approach. In this context, the use of genetic methods in order to facilitate the assessment of the structural diversity of organism communities has to be improved [39]. This section is not mandatory, but can be added to the manuscript if the discussion is unusually long or complex.
